# Development of a disposable paper-based thin film solid-phase microextraction sampling kit to quantify ketone body†

**DOI:** 10.1039/d4ra05907g

**Published:** 2024-10-11

**Authors:** Debsmita Mandal, Indrayani Dey, Chiranjit Ghosh

**Affiliations:** a Department of Biotechnology, Manipal Institute of Technology, Manipal Academy of Higher Education Manipal Karnataka 576104 India chiranjit.ghosh@manipal.edu; b Harvard Medical School 25 Shattuck Street Boston 02115 MA USA

## Abstract

Diabetes ketoacidosis (DKA) is a life-threatening complication and requires immediate medical attention in the case of diabetes subjects, especially in the case of type 1 diabetes mellitus. In the condition of DKA, the body produces an excess amount of ketone bodies after unregulated fat degradation, causing blood to become acidic and hampering the regular metabolic activities of the body. The current diagnostic technique for DKA condition is based on monitoring ketone bodies, especially β-hydroxybutyric acid, from human urine and blood samples. The detection of serum ketone bodies in pathology is sometimes limited due to false positive results and the lack of standardization for precise quantification of analytes. In this study, a paper-based patch operating on the thin film solid-phase microextraction (TF-SPME) principle was developed and it was coupled with gas chromatography-mass spectrometry for simple quantification of β-hydroxybutyric acid (BHB) ketone body from a phosphate-buffered saline matrix. To fabricate the paper-based TF-SPME patches, a regular A4 sheet paper sheet was utilized as the substrate and uniform coating by multiwalled carbon nanotubes (MWCNT), polydimethylsiloxane (PDMS) and divinyl benzene (DVB) compounds was performed with an automatic film applicator. The 70 μm paper-based coated sheet was trimmed into 4 cm × 1 cm dimension pieces to obtain multiple patches from a single sheet. Extraction of the BHB ketone body into the closed vials was performed by exploiting the individual DVB/PDMS and DVB/CNT/PDMS paper patches followed by desorption with acetonitrile before quantification by gas chromatography-mass spectrometry analysis. Our study showed that the BHB extraction efficiency of DVB/PDMS-coated patches was higher than that of DVB/CNT/PDMS. The outcome showed a good linearity (*R*^2^ = 0.99) within the 500–20 000 ng mL^−1^ concentration range of BHB by paper-based DVB/PDMS patches. This study demonstrated the feasibility of utilizing simple, cost-effective paper-based disposable TF-SPME patches as a sampling kit for future screening of diabetes ketoacidosis without the need for prolonged traditional sample preparation in pathology.

## Abstract 2

Illustration for determination of β-hydroxybutyric acid ketone body by paper-based thin film solid-phase microextraction patches.

## Graphical abstract

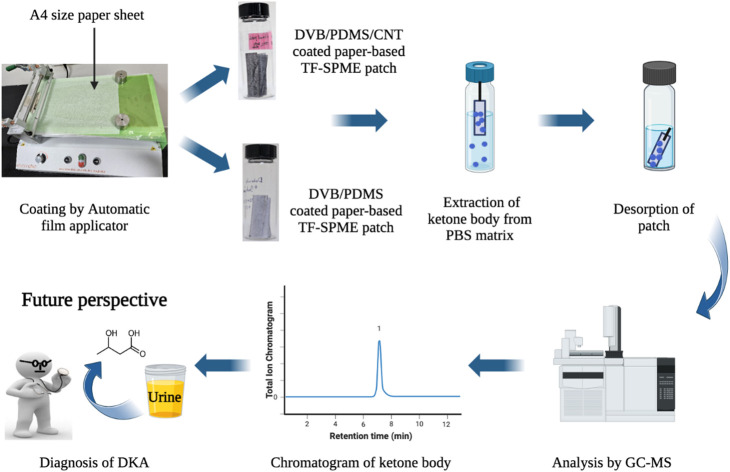

## 1. Introduction

Diabetes ketoacidosis (DKA) is a serious medical condition in diabetes individuals, primarily type 1 diabetes and needs prompt medical attention to avoid diabetes coma or even death. DKA happens when the body fails to produce sufficient insulin to utilize glucose as a primary source of energy, causing the enhancement of non-insulin-dependent fat metabolism as an alternate source of energy required for regular physiological activities. The unregulated rate of fat breakdown during DKA produces an excess amount of ketone bodies (β-hydroxybutyric acid, acetoacetate and acetone) as the by-products and these ketone bodies accumulate into blood, resulting in a decrease of blood pH (<pH 7.3). The presence of excess ketone bodies may cause the blood to become toxic and finally, hampers the regular metabolic functions in the body.^1,2^ The ketone body metabolites are excreted through sweat, urine and exhaled breath, appearing as free gases, aerosols, or both. In healthy subjects, the normal levels of ketone bodies in serum are below <0.5 mmol L^−1^, and more than 1.0 mmol L^−1^ is considered hyperketonemia, whereas in ketoacidosis, it has been reported to increase beyond 3.0 mmol L^−1^. To ensure the DKA state, several pathology tests, including blood glucose test (>250 mg dL^−1^), ketone body tests in blood and urine, serum bicarbonate test (below 18 mmol L^−1^), arterial blood gas measurement for pH (<pH 7.3), and anion gap (above 16 mmol L^−1^) are usually performed.^3^ However, ketone body levels are majorly measured to confirm the DKA state. During the last few years, several diagnostics tests were developed for the determination of ketone bodies from the human body.

Bayer Ketostix reagent strips are available to quantify acetoacetate in urine from human subjects. However, the chances of false negatives cannot be ignored from the mentioned test.^4^

Aneesh Koyappayil *et al.* proposed β-hydroxybutyrate dehydrogenase modified Ti_3_C_2_T_*x*_ MXene nanocomposite for estimation of β-hydroxybutyrate from serum samples.^5^ In further study, researchers proposed β-hydroxybutyrate dehydrogenase-modified biosensor and electrochemical paper-based analytical device (pop-up-EPAD) for measuring BHB from blood samples. Although the results were promising, it needed a blood sample.^6^ Although finger-prick-based blood collection is available, the random disposal of test strips after regular household use increases the chance of spreading blood-borne infectious pathogens in the environment. To simplify the ketone body detection, investigators proposed a NAD-dependent dehydrogenase-based wearable sensor to monitor BHB levels in urine and sweat.^7^ Further research is required to validate the technique on a large population. Hilary A. Byrne *et al.* proposed an electrochemical sensor to quantify the blood ketone bodies.^4^ Furthermore, laboratory-based ketone body tests are available. However, the application of regular pathology techniques is not practical in case of emergencies due to the prolonged sample preparation techniques before analysis. In the case of most studies, investigators mainly monitored the beta-hydroxybutyrate ketone body as estimation of only acetoacetate may underestimate the ketone body level during diagnosis of DKA. Therefore, there is a precise need for the development of a potential technique to estimate the ketone bodies for early diagnosis of DKA to prevent possible complications associated with the disease.

Solid-phase microextraction (SPME) is a well-known potential sample preparation technique for fast quantification of volatile and semi-volatile compounds. It was developed by Pawliszyn and his co-workers in the 1990s and revolutionized the field of analytical chemistry as it requires a minimum amount of solvents during sample preparation and minimizes the negative ecological effect of harmful chemicals, leading to a step towards green chemistry.^8,9^ The applications of SPME are widespread and are successfully used in various industries, such as pharmaceuticals, food, forensics, environmental analysis, drug monitoring *in vivo* and *in vitro*, fragrance analysis, *etc.*^10–18^ SPME technique was also employed for analysis of biological samples such as extraction of volatile organic compounds (VOCs) from different biological matrices, saliva analysis for prohibited substances, extraction and profiling of VOCs in case of oral cancer, urine analysis for the determination of estrogen, detections of steroid hormones in plasma, for metabolomic profiling of steroidal hormones from urine samples, peptide sampling and also for biomonitoring of organophosphate flame retardants (OPFRs) in urine.^19–26^

The principle of SPME is mainly based on the equilibrium partition theory between the sample matrix and the SPME fibre. Thin-layered fused silica-coated SPME fibre acts as an adsorbent surface for the extraction phase.^27^ Due to the low surface area and fragile nature of SPME fibre, thin-film solid-phase microextraction (TF-SPME) was developed as another geometry of SPME. The large surface area and flexible nature of TF-SPME membranes help to extract more analytes than the traditional SPME fibre. However, the operating principle of SPME is similar to that of TF-SPME. During the extraction process, the TF-SPME membrane is exposed to the sample matrix for a certain time to capture the analytes and finally, the membranes are desorbed for mass transfer from membrane to analyzer.^28^ The limited surface area of SPME-fibre reduces the extraction efficiency. Compared to SPME fibre, TF-SPME offers greater surface area, faster analyte collection, and more sensitivity.^29–31^

Recently development of paper-based analytical devices (PAD) has gained a lot of momentum in environmental and biomedical fields.^32^ Paper substrate provides a unique platform as it is cost-effective, easy to use, lightweight, sterile in nature, bio-compatible and easily disposable.^33–35^ Furthermore, its hydrophilic nature can be altered by coating with the hydrophobic materials to achieve different levels of hydrophobicity.^36^ PAD has extensively been utilized in surface-enhanced Raman spectroscopy (SERS) for the preconcentration and on-spot analysis of analytes such as the detection of sulfur dioxide (SO_2_) in wine, volatile aldehydes as biomarkers in lung cancer and the case of spot-on bioassays.^36–38^

In this study, the paper-based TF-SPME patches (pTF-SPME) were designed as a disposable sampling tool for the quantification of the ketone body from the PBS matrix. Mainly two individual types of patches were used, DVB/PDMS and DVB/CNT/PDMS, for extraction of β-hydroxybutyric acid and finally desorbed in gas chromatography-mass spectrometry for precise quantification of the ketone body. Therefore, this study may be useful for the fabrication of pTF-SPME for future diagnosis of diabetes ketoacidosis without the need for any blood test.

## 2. Materials and methods

### 2.1 Materials

Regular A4 sheet paper (Copy Gold A4, GSM-75, size 21 × 29.7 cm) was used as supporting material for fabricating the paper-based TF-SPME. The compounds, including polydimethylsiloxane (PDMS), multi-walled carbon nanotube (MWCNT) of 10 μm and divinyl benzene (DVB) monomers (80%)-technical grade, were purchased from Sigma Aldrich. Ketone body – DL-3-hydroxybutyric acid was purchased from TCI. Acetone and acetonitrile – ACN (99%) extra pure, 2,2-azobisisobutyronitrile – AIBN (98%), and absolute alcohol were purchased from LOBA Chemie. The hexane was obtained from Merck. Cotter pins for holding TF-SPME patches were procured from the local market. The 40 mL glass vials with septum & cap were purchased from Supelco. Tri-neck round bottom flask and other glass items were obtained from Borosil. PBS (pH 7.5) was prepared using 0.2 M NaH_2_PO_4_ (LOBA Chemie) and 0.2 M Na_2_HPO_4_ (LOBA Chemie) and diluted with distilled water. The stock solution of 100 000 ng mL^−1^ BHB was prepared using PBS and stored at 4 °C.

### 2.2 Instruments

Gas chromatography-mass spectrometer (GC-MS-QP2020 NX SHIMADZU, Shimadzu Corp., Tokyo, Japan) equipment was used for the measurement of BHB from the PBS matrix. Here, the GC-MS was equipped with an SH-I-5Sil MS column with a 0.25 mm internal diameter and 30 m length. The helium gas was used as a carrier gas. The GC-oven temperature was fixed as follows: 50 °C for 2 min, 10 °C up to 170 °C and hold for 1 min, 6 °C up to 230 °C and hold for 1 min and finally 15 °C up to 320 °C and hold for 2 min. The injection temperature was set at 280 °C in splitless injection mode and the flow rate of the helium stream was kept at 1.20 mL min^−1^.

To coat the TF-SPME membrane, an automated film applicator was used Elcometer 4310 (Aimil). The magnetic stirrer of model REMI-2MLH was purchased from REMI. The vortex model REMI-CM-101 plus and shaking incubator of model REMI-CIS-18plus were purchased from REMI. The centrifuge was procured from Eppendorf.

### 2.3 Statistical method

OriginPro 2024b (Origin Lab Corporation, USA) software was utilized for statistical analyses of the data. The data was expressed as mean ± SE (standard error). To depict the reproducibility of data at various concentration ranges, the calibration curves were plotted based on three repetitions at each data point. The fittings equations were obtained from the linear fittings plot of the calibration curves. The linear regression data were calculated from the software.

### 2.4 Synthesis of DVB particles

**Fig. 1:**
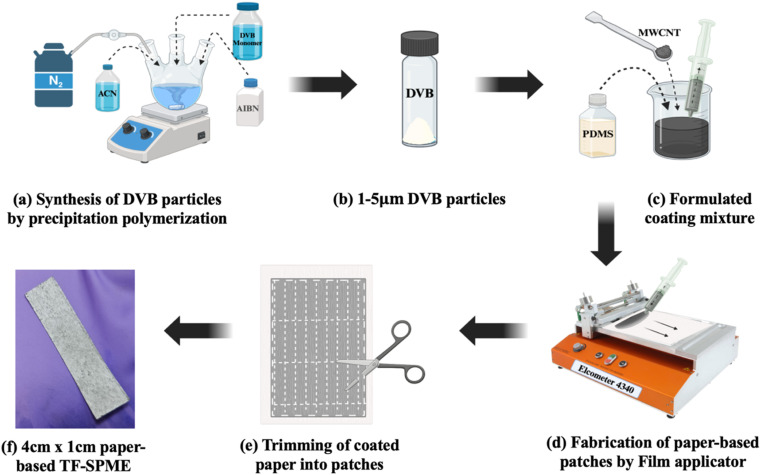
Procedure for the fabrication of paper-based TF-SPME by film applicator.

DVB particles were synthesized using the precipitation polymerization process. In this method, DVB monomers were polymerized to form DVB particles. In a tri-neck round bottom flask with a mechanical stirrer, 200 mL of acetonitrile (ACN) was added, and then nitrogen gas was purged for 2 h, as shown in Fig. 1a. Then, 5 mL of DVB monomers were added to ACN, followed by a mixing of 300 mg of 2,2-azobisisobutyronitrile (AIBN) as the initiator of the reaction. The mixture was heated at 70 °C to initiate the free radical polymerization process and then kept for 24 h at 100 rpm speed for the polymerization to occur. Then, the reaction mixture was collected through centrifugation at 10 000 rpm for 30 min. The DVB particles were washed with ethanol three times, and then they were kept for drying under sterile conditions at normal room temperature and stored in a closed vial as seen in Fig. 1b.

### 2.5 Preparation of DVB/PDMS/CNT and DVB/PDMS coating mixtures

To prepare the DVB/PDMS/CNT mixture, 1 g of DVB, 3.59 g of PDMS, 0.06 g of CNTs, and 8.52 mL of hexane were added to a beaker as shown in Fig. 1c and kept on a magnetic stirrer for 24 h to get a homogenous mixture. The beaker was covered with aluminium foil, and tiny holes were made in the foil so that hexane could evaporate. In the case of only DVB/PDMS coating, 0.973 g of DVB particles were weighed in a beaker, and then 3.74 grams of PDMS and 13 mL of hexane were added and the same procedure was followed as mentioned above.

### 2.6 Sample preparation for BHB

First, 25 mL of 100 000 ng mL^−1^ of BHB stock solution was prepared using phosphate buffer saline (PBS) as a matrix from pure BHB standard and kept at 4 °C temperature in a 40 mL vial (Sigma Aldrich – Supelco® clear vial). Concentrations of 500 ng mL^−1^, 1000 ng mL^−1^, 5000 ng mL^−1^, 10 000 ng mL^−1^ and 20 000 ng mL^−1^, with each having three replicates, were prepared from the stock solution and vortexed thoroughly to get a homogenous solution and stored at 4 °C temperature.

### 2.7 Fabrication of paper-based TF-SPME patches

To fabricate the paper-based TF-SPME patches, a clean A4 sheet was taken and coated with previously prepared coating mixtures with the help of the automated coating instrument – Elcometer 4310, as seen in Fig. 1d, and the coating thickness was kept at 70 μm, and the minimum speed was kept to get an evenly coated membrane. The coated A4 paper was dried in an aseptic condition to avoid any contamination. The same procedure was followed to coat another side of the same paper with the same mixture. After drying the paper, it was trimmed into 4 cm (L) × 1 cm (W) patches shown in Fig. 1f and CNT/DVB/PDMS and DVB/PDMS coated patches were made and kept in air-tight vials.

### 2.8 Extraction of BHB by TF-SPME

**Fig. 2:**
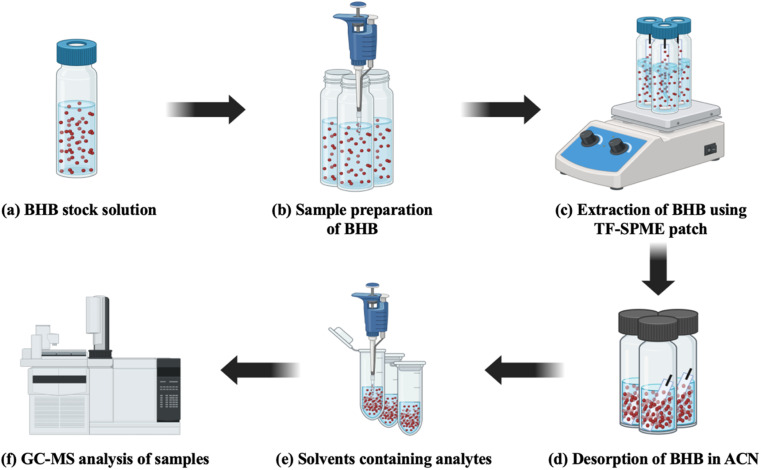
Illustration of extraction of BHB using paper-based TF-SPME patches.

For the extraction of BHB by TF-SPME, 15 paper-based 4 cm (L) × 1 cm (W) TF-SPME patches were chosen from DVB/PDMS/CNT coated patches and each of them was attached to a cotter pin and with the help of cotton thread and cello tape it was suspended from the top inside of the vial (Sigma Aldrich – Supelco ® clear vial). Each vial containing a directly immersed paper-based TF-SPME patch and a smaller-sized magnetic bead inside was kept on a magnetic stirrer for 4 h for the proper extraction of BHB from the sample matrix to the patch at 30 °C as seen in Fig. 2c. After the extraction phase, the patches were transferred into clear transparent vials after washing with 1 mL of ACN. Vials were vortexed 3 times and kept for 1.5 h for proper desorption at room temperature. Then, 1 mL of ACN-containing analytes was transferred into Eppendorf tubes (2 mL) and sealed properly with parafilm before sending those for analysis in GC-MS-QP2020 NX SHIMADZU, ShimadzuCorp (Tokyo, Japan) as depicted in Fig. 2d–f. The same procedure was followed for the extraction and desorption of BHB using DVB/PDMS-coated patches.

## 3. Results and discussion

### 3.1 Characterization of DVB particles

**Fig. 3:**
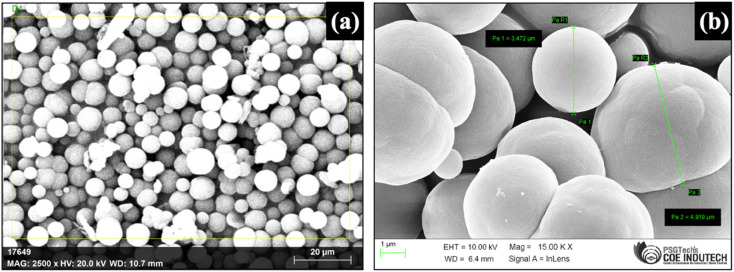
DVB particles synthesized by precipitation polymerization. SEM images (a) at magnification 2500× at 20.0 kV [CARL ZEISS (USA), model: Sigma with Gemini column, resolution 1.5 nm] and (b) particle size of DVB polymer (1–5 μm).

A new method involving an easy disposable paper-based TF-SPME patch compatible with GC-MS was developed for the determination of BHB from the PBS matrix. The DVB particles that were synthesized in the laboratory using DVB monomer by precipitation polymerization method were characterized by SEM, SEM-EDAX and FT-IR techniques. To visualize the morphological features, SEM (Scanning Electron Microscope) was used. Fig. 3a and b show the SEM images of synthesized DVB particles of approximately 1–5 μm in size.

SEM-EDAX (Energy-dispersive X-ray Analysis) analysis was done to check the compositions of the elements in the synthesized particles qualitatively and quantitively. It was observed that this polymer is mainly composed of carbon and oxygen atoms which are present as elements at 89.74% and 10.26% respectively (Fig. S1†).

**Fig. 4:**
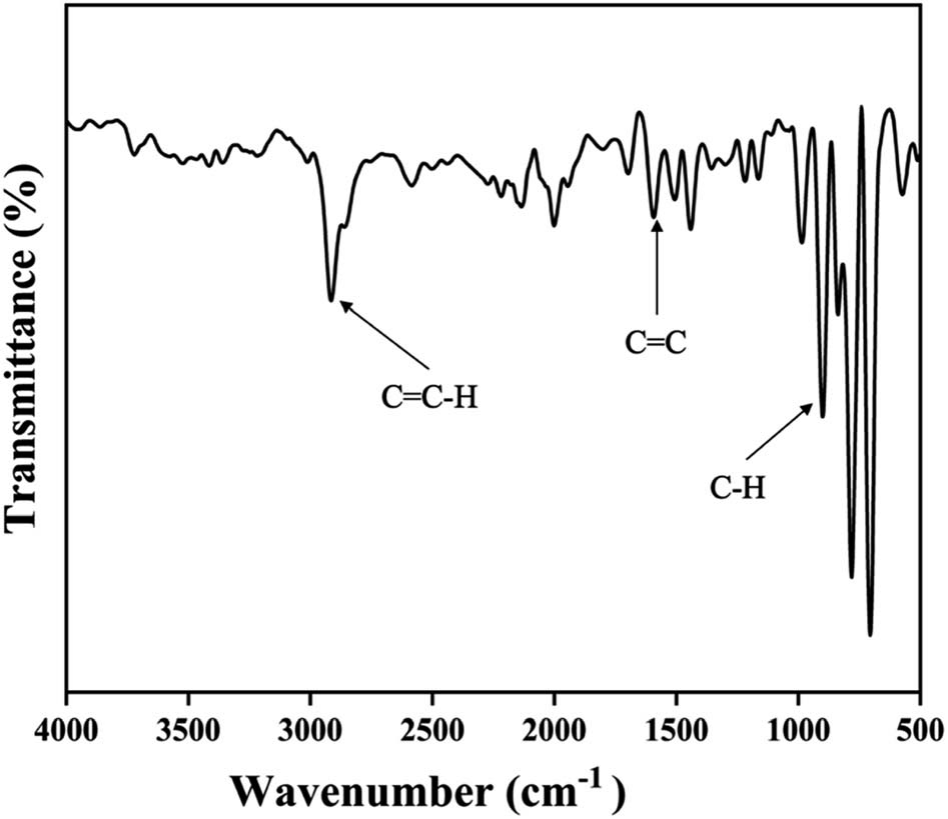
FT-IR spectrum of DVB particles synthesized by precipitation polymerization.

FT-IR (Fourier Transform Infrared Spectroscopy) spectrum has been shown in Fig. 4. The peaks around 3000 cm^−1^, 1600–1500 cm^−1^, and 900 cm^−1^ were respectively assigned to aromatic C=C–H, stretching, C=C stretching, and aromatic C–H bending.

### 3.2 Stability of paper-based TF-SPME

#### 3.2.1. Thermogravimetric (TGA) analysis

**Fig. 5:**
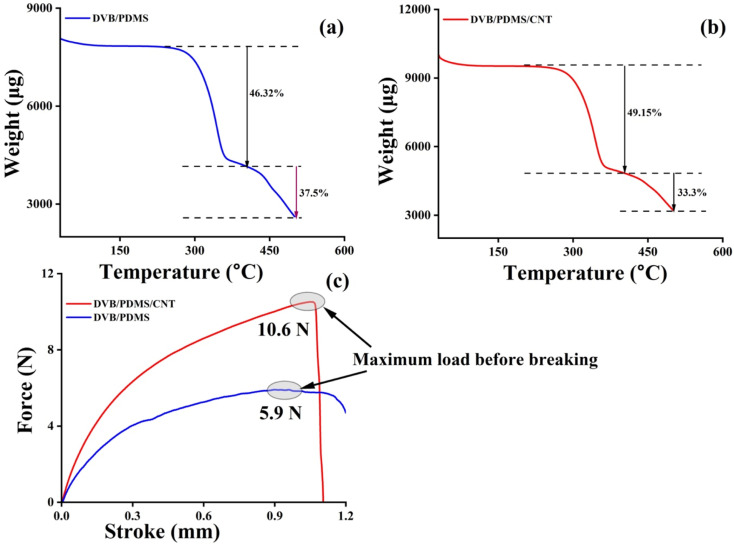
Thermal and mechanical stability check by TGA (a) and (b) and UTM (c) experiments with DVB/PDMS/CNT and DVB/PDMS coated paper patches for stability test.

Thermogravimetric analysis was conducted with Hitachi STA7200 equipment. The operation was performed at a temperature range from 30 °C to 500 °C with a heating ramp temperature rate of 5 °C min^−1^ under nitrogen condition. The sample masses of 8 mg and 10 mg were taken for performing the test with DVB/PDMS (Fig. 5a) and DVB/PDMS/CNT-coated (Fig. 5b) patches respectively. The first thermal degradation temperature for both paper-based patches was observed in the range of 230–350 °C, suggesting the considerable thermal stability of our fabricated microextraction patches for regular use.

#### 3.2.2. UTM analysis for stability/tensile testing

To check the mechanical stability of the proposed paper-based TF-SPME patches, the tensile test was performed using the UTM machine by Shimadzu-EZ-SX equipment. The UTM data (Fig. 5c) depicted that the CNT-coated patch (DVB/PDMS/CNT) endured more mechanical strength than the non-CNT-coated patch (DVB/PDMS) before tearing of the patches. The UTM results demonstrated that the DVB/PDMS/CNT-coated paper patch was more mechanically stable than the DVB/PDMS patches. The paper TF-SPME is quite stable for our application for this study.

### 3.3 Optimization of method by paper-based TF-SPME

#### 3.3.1. Effect of pH

**Fig. 6:**
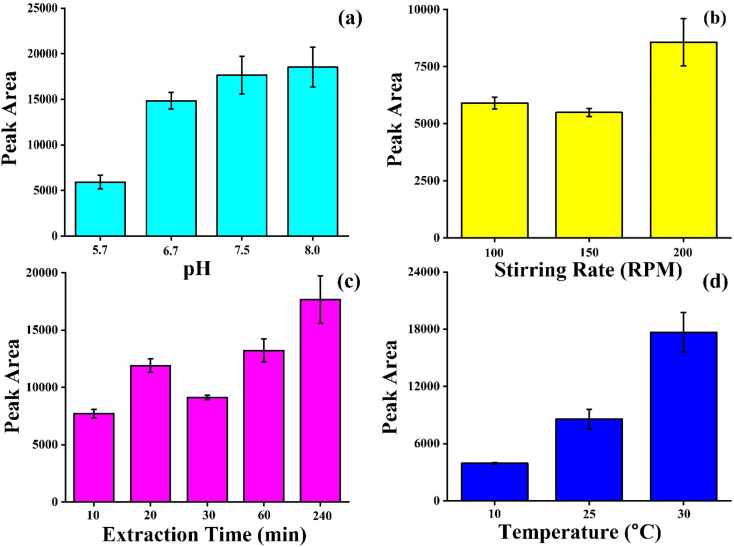
Optimization of BHB extraction method by paper-based TF-SPME patches in BHB matrix at various (a) pH, (b) stirring rate, (c) extraction time and (d) temperature.

The effect of pH on the performance of paper-based TF-SPME was investigated by individual experiment of BHB solutions across a pH range 5.7–8. The TF-SPME patches were used for extraction of BHB from a standard solution with 5000 ng mL^−1^ concentration of BHB. The extraction efficiency of our fabricated microextraction patch was observed to be enhanced with an increase in pH. However, there was no significant difference in extraction efficiency between pH 7.5 and pH 8 (Fig. 6a). It is also noteworthy that exposure of cellulose paper in acidic media may facilitate the hydrolysis of the paper, whereas the high pH may be responsible for losing the mechanical strength of microextraction tool and enhancement for chance of swelling of the patches during experiments. Therefore, we have selected the optimal pH as 7.5 for our study.

#### 3.3.2. Effect of stirring rate

Extraction is a dynamic process where the adsorption and desorption are two continuous processes during the study by TF-SPME patches. Enhancement of stirring rate may increase the movement of analytes from bulk to the surface of TF-SPEM tool. It also helps to reach the equilibrium quickly and simultaneously reduce the extraction time. However, high stirring speed may damage the patches during the experiments. The effect of stirring rate on extraction efficiency of our proposed method was studied with different stirring speeds including 100 rpm, 150 rpm and 200 rpm. The Fig. 6b illustrated that the maximum extraction efficiency by our microextraction patch was observed at 200 rpm from 5000 ng mL^−1^ BHB standard solutions. Therefore, 200 rpm was selected as the optimum stirring rate for this study.

#### 3.3.3. Effect of extraction time

It is important to optimize the extraction time for effective preconcentration of BHB from sample matrix to microextraction tool as it is associated with the amount of analytes extracted and finally performance of the technique. Fig. 6c shows the extraction efficiency of 5000 ng mL^−1^ BHB standard solutions at different time intervals – 10 min, 20 min, 30 min, 60 min, and 240 min. The extraction efficiency reached at the highest level at 240 min. The diffusion of analytes from samples matrix to sorbent materials on paper patches increases with time. To obtain potential efficiency, the extraction time of 240 min was selected for this study.

#### 3.3.4. Effect of temperature

Temperature plays a vital role in influencing diffusion rate analyte–sorbent interaction during the extraction of BHB by paper patches. The high temperature may increase the movement of analytes from sample matrix to adsorption phase of TF-SPME and may reduce the equilibrium time and increase the adsorption process. However, a very high temperature may also increase the desorption rate of analytes from sorbent to sample solution. Here, the effect of temperature on BHB extraction efficiency was studied at three different temperatures (10 °C, 25 °C, 30 °C). The maximum extraction efficiency was observed at 30 °C (Fig. 6d) from 5000 ng mL^−1^ BHB standard solution by the proposed TF-SPME patches. Here, further increase of temperature was not performed as it was important to us to check the feasibility to utilize a simple paper-based TF-SPME patch for use in room temperature for regular application including clinical settings in future.

### 3.4 GC-MS analysis

**Fig. 7:**
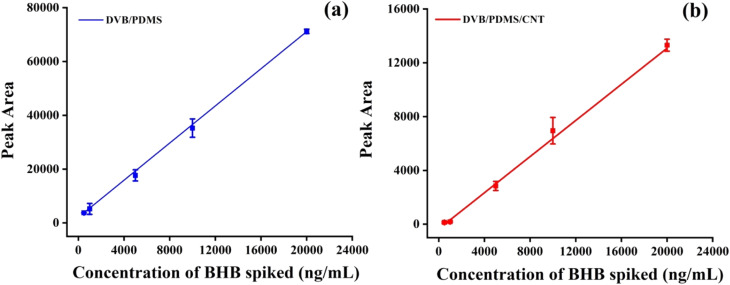
(a) Calibration curve of BHB extraction by only DVB/PDMS-coated paper-based TF-SPME patch and (b) with DVB/PDMS/CNT coated paper-based TF-SPME patch. The fitting equations were *y* = 3.45*x* + 2042.8 (DVB/PDMS) and *y* = 0.67*x* − 349.9 (DVB/PDMS/CNT).

To derive the quantity of BHB extracted by the TF-SPME patch, the GC-MS responses were recorded with the BHB quantities spiked into the PBS matrix following the extraction of BHB by microsorbent patches. In the chromatogram (Fig. S2†) containing BHB, the peak was seen at a retention time of 6 min. The amount of BHB extracted by patches was calculated from the calibration curve after direct immersion of patches into BHB-containing test vials with multiple concentrations. The pH of the PBS matrix was kept at 7.5. Fig. 7a demonstrates the five-point calibration curves utilizing DVB/PDMS and DVB/PDMS/CNT (Fig. 7b) coated patches. The curves exhibited good linearity with *R*^2^ values of around 0.99 of a spiking concentration range of 500–20 000 ng mL^−1^ of BHB standard solution. The theoretical value of LOD and LOQ for the DVB/PDMS coated patches were calculated at approximately 11.3 ng mL^−1^, and 34.4 ng mL^−1^. Multiple replicates showed that the data were reproducible. The results showed that DVB/PDMS coated TF-SPME patch extracted more BHB at a certain time than DVB/CNT/PDMS. This may be the reason that BHB is moderately hydrophilic in nature with log *P* < 0, and it can dissolve in human blood and urine matrix. MWCNT utilized here is hydrophobic in the coating recipe. Although CNT has high thermal, chemical and mechanical stability with high extraction efficiency, the carbon backbone structure facilitates it to extract hydrophobic compounds efficiently. The fittings equation derived from the DVB/PMDS calibration curve was *y* = 3.45*x* + 2042.8. The unknown concentration of BHB can be calculated utilizing the fittings equation. To check the accuracy of the proposed technique, we spiked various concentrations of BHB in the sample matrix and calculated the recovery and accuracy of the tests. We observed that the accuracy of the proposed techniques varies from almost 90% to 99% (Table 1), suggesting the applicability of the proposed method for the practical application.

**Table 1:** Accuracy test after spiking BHB into PBS matrix and calculation of recovery as well as accuracy from the fittings equation associated with DVB/PDMS-coated paper TF-SPME

Sample	Spiked	Recovery (as per proposed method)	Accuracy (%)
BHB concentration (ng mL^−1^)	5000	5131	∼98.4
	10 000	11 125	∼89.7
	20 000	19 817	∼99.1

## 4. Conclusion

The present study described the fabrication of the disposable paper-based patch for quantifying 3-hydroxybutyric acid, a ketone body usually used to diagnose diabetes ketoacidosis state. The data demonstrated the feasibility of a polymer-coated disposable sampling tool for the estimation of 3-hydroxybutyric acid from the phosphate-buffered saline matrix (a similar matrix to the urine sample), thus obviating the need for invasive blood samples and prolonged sample preparation procedures during traditional methods. We observed good accuracy of our proposed technique during the method validation. The proposed working equation derived from the calibration curve for determining the ketone body may largely facilitate the rapid screening of DKA in hospital and regular clinical settings. The TF-SPME tool may act as an emerging tool for precontraction of BHB from biological samples and analysis of trace quantity of the ketone body without need for tedious method development. In addition, the experimental results showed good extraction efficiency and reproducibility, suggesting for feasibility of this proposed technique for routine clinical application. Furthermore, the paper-based DVB/PDMS coated TF-SPME is disposable and easy to handle as compared to SPME fibre, suggesting the possibility for extraction of large quantities of analyte from the clinical samples. This study demonstrated the future perspective for utilization of the microextraction patch as a sampling kit for the analysis of BHB for diagnosis of DKA. There are several advantages in use of paper-based TF-SPME for quantification of BHB, including the biodegradability and environmental friendliness, ease of fabrication, requirement of low amount of solvent and simplified sample handling for this study. However, further research is required to validate the method in real biological samples. It needs more investigations for enhancement of mechanical strength of the paper-based polymer-coated TF-SPME to avoid the possibility for tearing during the experiments. As the selection of sorbent materials is based on the compatibility on paper-substrate, the selectivity of this technique may be further improved through incorporation of suitable polymers and nanoparticles. Although there are several disadvantages of this technique, this technique may be adopted due to its simplicity and potential in use as a disposable analytical tool for analysis of clinical samples. In future research, it may be possible to couple the paper-based TF-SPME sampling kit with portable GC-MS for real time determination of the ketone body.

## Data availability

All the authors confirm that there is no supplementary data available for this original article. However, the data will be shared after getting the request.

## Conflicts of interest

All authors acknowledged no conflicts of interest.

## Supplementary Material

RA-014-D4RA05907G-s001
