# Supplementary material for: Development of a disposable paper-based thin film solid-phase microextraction sampling kit to quantify ketone body
Source: RSC Adv. 2024 Oct 11;14(44):32230–8. doi: 10.1039/d4ra05907g (PMC11469451; doi:10.1039/d4ra05907g)
Supplement: RA-014-D4RA05907G-s001 [file RA-014-D4RA05907G-s001.pdf]

Supplementary Information (SI) for RSC Advances.
This journal is © The Royal Society of Chemistry 2024

**Supporting Information:**

# Development of disposal paper-based thin film solid-phase microextraction sampling kit to quantify the ketone body

Debsmita Mandal[a], Indrayani Dey[a] and Chiranjit Ghosh[a,b]*

[a]*Department of Biotechnology, Manipal Institute of Technology, Manipal Academy of Higher Education, Manipal, Karnataka 576104, India*

[b]*Harvard Medical School, 25 Shattuck Street, Boston, 02115, MA, USA*

*__Corresponding Author:__ Chiranjit Ghosh **Email:** **chiranjit.ghosh@manipal.edu**

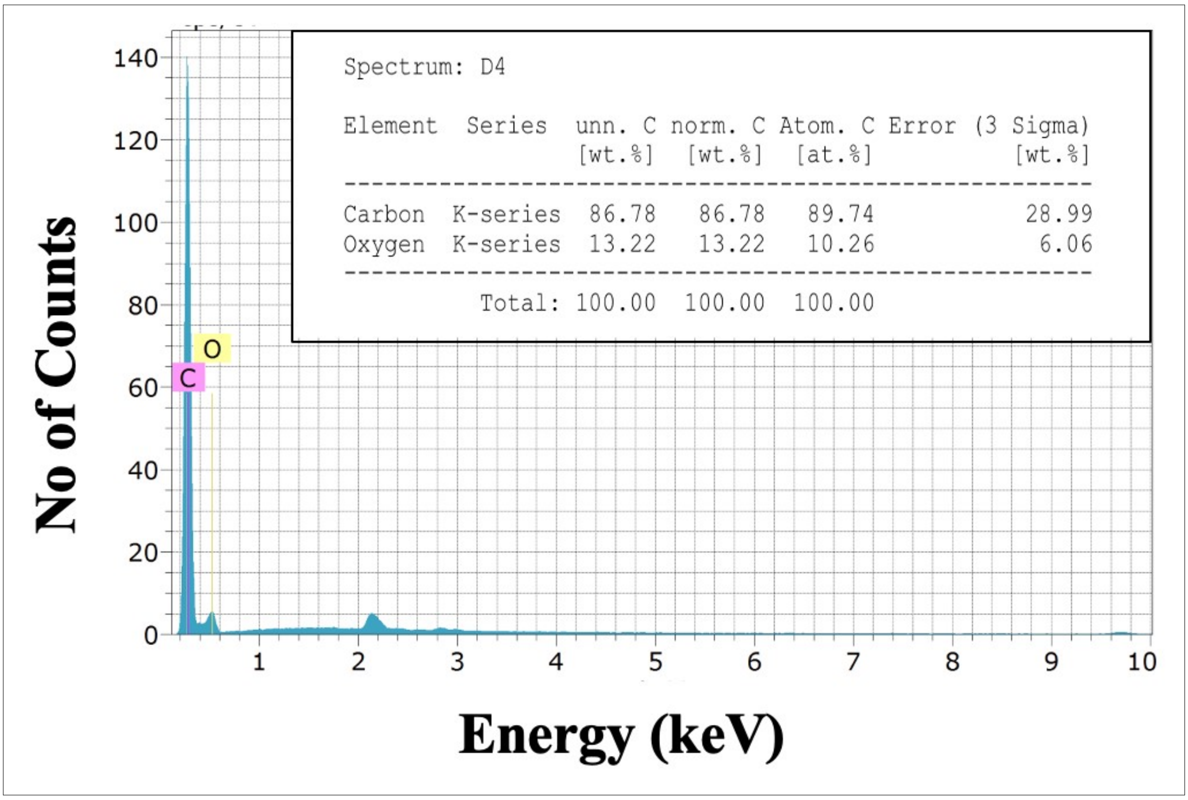


Fig.S1. SEM-EDAX image and data of synthesized DVB particles [BRUKER (German), model: Nano XFlash Detector]

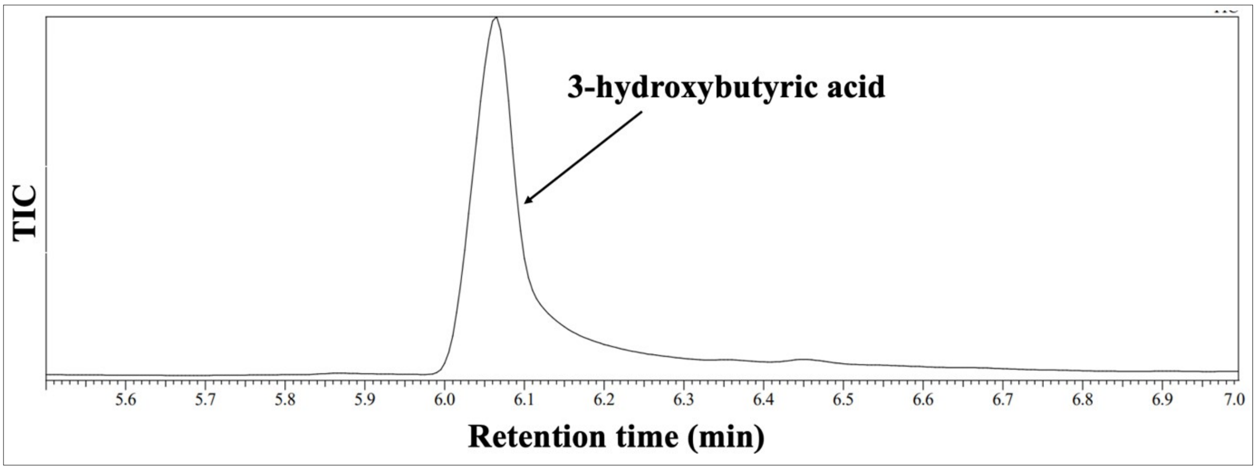


Fig.S2. Chromatogram of BHB at RT 6min in PBS matrix by paper-based TF-SPME
